# Behavioral signature of intraspecific competition and density dependence in colony-breeding marine predators

**DOI:** 10.1002/ece3.754

**Published:** 2013-09-12

**Authors:** Greg A Breed, W Don Bowen, Marty L Leonard

**Affiliations:** 1 Department of Biology, Dalhousie University1355 Oxford Street, Halifax, Nova Scotia, B3H 4J1, Canada; 2 Bedford Institute of Oceanography1 Challenger Drive, Dartmouth, Nova Scotia, B2Y 4A2, Canada

**Keywords:** Animal movement, compensatory population regulation, correlated random walk, foraging ecology, juvenile mortality, marine mammal, seal, switching state-space model

## Abstract

In populations of colony-breeding marine animals, foraging around colonies can lead to intraspecific competition. This competition affects individual foraging behavior and can cause density-dependent population growth. Where behavioral data are available, it may be possible to infer the mechanism of intraspecific competition. If these mechanics are understood, they can be used to predict the population-level functional response resulting from the competition. Using satellite relocation and dive data, we studied the use of space and foraging behavior of juvenile and adult gray seals (*Halichoerus grypus*) from a large (over 200,000) and growing population breeding at Sable Island, Nova Scotia (44.0 ^o^N 60.0 ^o^W). These data were first analyzed using a behaviorally switching state-space model to infer foraging areas followed by randomization analysis of foraging region overlap of competing age classes. Patterns of habitat use and behavioral time budgets indicate that young-of-year juveniles (YOY) were likely displaced from foraging areas near (<10 km) the breeding colony by adult females. This displacement was most pronounced in the summer. Additionally, our data suggest that YOY are less capable divers than adults and this limits the habitat available to them. However, other segregating mechanisms cannot be ruled out, and we discuss several alternate hypotheses. Mark–resight data indicate juveniles born between 1998 and 2002 have much reduced survivorship compared with cohorts born in the late 1980s, while adult survivorship has remained steady. Combined with behavioral observations, our data suggest YOY are losing an intraspecific competition between adults and juveniles, resulting in the currently observed decelerating logistic population growth. Competition theory predicts that intraspecific competition resulting in a clear losing competitor should cause compensatory population regulation. This functional response produces a smooth logistic growth curve as carrying capacity is approached, and is consistent with census data collected from this population over the past 50 years. The competitive mechanism causing compensatory regulation likely stems from the capital-breeding life-history strategy employed by gray seals. This strategy decouples reproductive success from resources available around breeding colonies and prevents females from competing with each other while young are dependent.

## Introduction

Intraspecific competition is a primary mechanism of density-dependent population regulation (Nicholson 1954; May et al. 1974; Furness and Birkhead 1984). The type of intraspecific competition (e.g., scramble, contest, interference) and the limiting resource, however, can have major effects on the functional response of a population as it approaches carrying capacity (K) (May et al. 1974). Competition is often for food resources, but it can also be for space, breeding sites, territories, or other vital resources.

Although intraspecific competition is the most common cause of density dependence, conclusive demonstration of such competition usually requires removal of individuals to relax competition for suspected limiting resources. Where such manipulations cause competing population segments to move into a previously occupied niche, experience increased survival, show better condition, or have increased fecundity, intraspecific competition is occurring (Dayton 1971; Paine 1984). Such manipulations are often impractical in free-living populations, and intraspecific competition usually must be inferred circumstantially (Hansen et al. 1999). Such inferences are often based on overlap or segregation in diets or space (e.g., Tinker et al. 2008a). Although the later may represent niche partitioning, differential response to predation risk, or other noncompetitive segregating mechanism, population growth accompanied with decreased performance or survival in one of the suspected competing groups more strongly suggests intraspecific competition.

Because of the difficulty of studying wild populations, much of our understanding of intraspecific competition and density-dependent population dynamics relies upon laboratory mesocosm experiments and population modeling (e.g., Bellows 1981). Using these approaches, Maynard-Smith and Slatkin (1973) and Bellows (1981) predicted that different kinds of intraspecific competition should give rise to different density-dependent functional responses as a population approaches K. Scramble competition, where all competitors are equally able to acquire a limiting resource, should cause overcompensatory regulation, with the potential for major population-level mortality or reproductive failures near K. When resources become limiting, the equal competitors all receive an inadequate ration and theoretically all starve. The number of starving animals can be far larger than the degree to which a population exceeds K.

Competition that results in one competitor or competing group being better able to acquire or defend a limiting resource (e.g., contest competition) should cause compensatory regulation. In this case, when resources become limiting, better competitors receive an adequate ration, and poorer competitors starve. The number which starve is thus directly proportional to the degree a population exceeds K. Compensatory regulation should result in a gradual decrease in population-level reproductive success and a gentle approach to K. Where enough behavior and population data are available, it should be possible to infer the type of intraspecific competition and predict the functional form of regulation experienced by a population.

Many marine birds, mammals, and reptiles breed at colonies, and intraspecific competition can be keen and develop rapidly or unexpectedly when resources around colonies fluctuate (Furness and Birkhead 1984; Schreiber and Schreiber 1984; Trillmich and Limberger 1985; Wanless et al. 2005). At the same time, colony-breeding marine species are often top predators and may also be of conservation concern. Understanding the population dynamics of such species can be extremely important for population conservation as well as overall ecosystem management.

**Plate 1:**
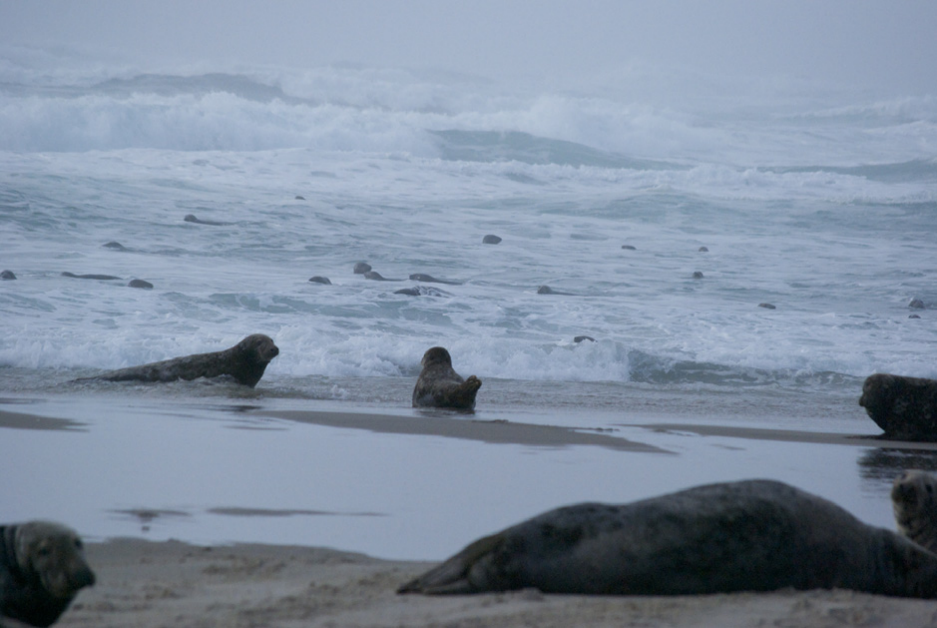
Gray seals in the surf at Sable Island, Nova Scotia.

Here, we examine evidence for intraspecific competition and its behavioral signature in animal movement patterns from a growing population of gray seals (*Halichoerus grypus*) in the Northwest Atlantic (Plate 1). This population breeds as a colony on Sable Island, Nova Scotia, and was historically suppressed by hunting and bounties at coastal haulouts and nearshore waters of eastern Canada. In the early 1960s, gray seals were considered rare, and the Sable Island population produced only a few hundred offspring annually. The population grew exponentially from 1962 until the early 2000s (Bowen et al. 2003), but more recently appears to have entered the deceleration phase of density-dependent logistic growth (Bowen et al. 2007, 2011).

The dramatic growth of gray seals has been accompanied by major changes in the structure and functioning of the Scotian Shelf ecosystem (Frank et al. 2005, 2011). There is also concern that gray seal predation on commercially important or rare fishes may cause serious harm to these stocks (e.g., Trzcinski et al. 2006; Benoît et al. 2011). Understanding the mechanism of density dependence will help predict future population trends, gray seal predation rates on fishes, gray seal impacts on the structure and stability of the Scotian Shelf ecosystem, and the results of potential management actions. More broadly, such information may help predict which types of behaviors and intraspecific interactions should be expected around marine animal colonies and how populations might respond to those interactions.

Our analysis focuses on differences in distribution and habitat usage among age and sex classes to gain insight into the forces influencing movement decisions and foraging behavior. To understand the context and cause of the differences, we synthesize our results within a larger body of data that includes changes in survival of juveniles, diet breadth, physiological limitations of juveniles, and prey distribution and quality.

Taken together, the evidence supports a number of potential segregating mechanisms. We present the case for several alternative hypotheses, including differential habitat preference, niche partitioning, and differential response to shark predation. Although our results do not conclusively exclude these noncompetitive segregating mechanisms, the pattern of habitat segregation is most consistent with intraspecific competition and we contend this competition is resulting in the currently observed density-dependent population growth. Finally, we discuss the potential impact of the hypothesized intraspecific competition on this population and predict the functional response of density-dependent regulation should be compensatory and not overcompensatory and compare this prediction to the observed population dynamic.

## Methods

### Data collection

In late May and early June 2004, twenty-four (12 male, 12 female) young-of-year (YOY), 15 adults (7 male, 8 female), and 6 subadults (2–3 years old; 4 male, 2 female) gray seals were captured on Sable Island, Nova Scotia (44.0^°^N 60.0^°^W). Seals were captured with handheld nets, anaesthetized with Telazol, and Argos satellite transmitters (Sea Mammal Research Unit model SRDL 7000) were attached with 5-minute epoxy as described in Breed et al. (2011a). The adults became part of a larger data set that included a total of 81 adults tagged between 1995 and 2005 (Breed et al. 2006), allowing for comparison of adult, subadult, and YOY at-sea movement.

### Movement and spatial behavior

Argos tracking data were analyzed with a behaviorally discriminating state-space model (SSM), which statistically reduces the influence of Argos location error, estimates locations at regular time-intervals, and infers behavioral states. We chose a time-step of 8 h or three points per day as this was approximately the observation frequency of our lowest quality tracks (see Breed et al. 2009, 2011b for more information on time-step selection and the effect of data quality on inference and model performance). The model explicitly estimated behavioral state by switching between two sets of parameters describing a correlated random walk (CRW). Displacements better fit by parameters of high first-order autocorrelation and turn angles near 0^°^ were nominally termed “transiting,” while displacements that were better fit by parameters of low autocorrelation and turn angles near 180^°^ were nominally termed “foraging.” Movements inferred as “foraging” cause animals to remain in a small area of ocean or are part of an area-restricted search. Such movement patterns are well documented to be associated with increased prey capture rates (Austin et al. 2006; Dragon et al. 2012). Transiting states were associated with rapid directional movement.

We used a two-state model for simplicity. Fitting two states can be performed relatively simply with fairly course spatial data and identifying two behavioral classes extracts considerable behaviorally relevant information. Inferring three or more states is less straightforward and usually requires higher quality behavioral data and often strong Bayesian priors (Breed et al. 2012; McClintock et al. 2012). Without such high-quality data (e.g., high spatiotemporal resolution GPS or triaxial accelerometry data), inferring three or more states usually encounters significant identifiably issues. The model was fit to demographically homogenous groups of up to 15 tracks so that short tracks could borrow power from longer tracks to infer behavioral states (see Breed et al. 2009 for details). The movement parameters themselves were similar across all groups, and this approach is validated in an analysis in the appendix of Breed et al. (2009), which shows that tracks fit individually had similar estimates of movement parameters and inferred behavioral states as those fit in groups. The SSM was implemented using the freely available software packages R and WinBUGS (Spiegelhalter et al. 2007; R Development Core Team 2012). Working code and implementation details of this behavior-switching SSM can be found in Breed et al. (2009, 2011b).

### Dive and time budget data

The 45 Argos tags deployed in 2004 collected diving data, but Argos bandwidth limited the data available to 3-h binned summaries of dive characteristics. Numerous dive characteristics were available, but we chose three key variables: maximum dive depth, mean dive duration, and haulout (i.e., time spent on land as indicated by wet/dry switches), which are most strongly limited by physiological immaturity. Dive data were used to investigate age-related differences in diving ability. Haulout data were compared to SSM foraging locations that occurred within 10, 20, and 30 km of shore as well as those beyond 30 km to produce time budgets of time spent foraging in areas of varying accessibility relative to haulout sites. These budgets were used to investigate displacement of juveniles from the most accessible foraging regions to more distant areas by adults and to validate the kernel overlap analysis, which we describe shortly. Time budgets and binned dive summary characteristics of demographic groups were statistically compared using mixed-effects models with either a log or logit link in the nlme package of R (R Development Core Team 2012).

### Spatial distribution and habitat use

Clumps of inferred foraging locations from YOY, adult males, and adult females, appear to occur in different areas on the Scotian Shelf. To test if these apparent differences were caused by chance or were real, we employed the kernel density overlap analysis described in Breed et al. (2006). In this analysis, we overlay the kernel densities of different groups and measure their areal overlap. To test if this overlap is larger or smaller than might be expected by chance, we compare the size of the overlap to the overlap of kernels calculated from two null groups. These null groups are produced by randomly assigning tracks to each of two groups to be compared instead of grouping them according to their age by sex demographic class. If the real overlap is smaller than a large set (95%) of randomly produced null overlaps, the lack of overlap is significant and biologically relevant.

The method was modified to include the behavioral state information available in switching SSM results. As our goal was to test for differences in foraging areas, kernels were constructed using only at-sea SSM inferred foraging locations, excluding transit locations and all locations within 2 km of shore. The size of the grid (100 by 100 nodes, with 10.75 km between nodes), the fixed kernel smoothing, and width parameters, as well as the choice of 98.5% density contour used to calculate the range overlaps, were the same as those used in Breed et al. (2006).

Kernels were computed for YOY, adult males, and adult females for each month from June to January. There were too few data to include subadults and too few data from YOY to compare with adults from February to May (Table 1). Areal overlap of the 98.5% density contour was calculated first for male versus female YOY to test for evidence of sexual segregation within this age group, then for adult males versus YOY and again for adult females versus YOY. Adult males versus adult females were not tested because the results of a similar analysis can be found in Breed et al. (2006).

**Table 1:** Sample sizes for the kernel density randomization analysis

	Number of Tracks			Number of Locations		
		
Month	Males	Females	YOY	Males	Females	YOY
Jun	14	14	24	364	509	1088
Jul	14	15	23	594	627	1017
Aug	14	15	23	473	515	960
Sep	26	25	19	622	653	843
Oct	32	31	17	1158	1363	895
Nov	31	31	17	1367	1989	862
Dec	25	28	14	1027	1480	561
Jan	18	24	13	186	496	475
Total	43	38	24	7022	9538	7239

“Number of Tracks” is the number of animals with locations in a given month. Animals whose tracks spanned more than one month were counted in the sample of each month the track spanned. “Number of Locations” is the number of at-sea SSM inferred foraging locations recorded in a given month; inferred transiting, haulout, and locations within 2 km of shore were excluded.

**Figure 1:**
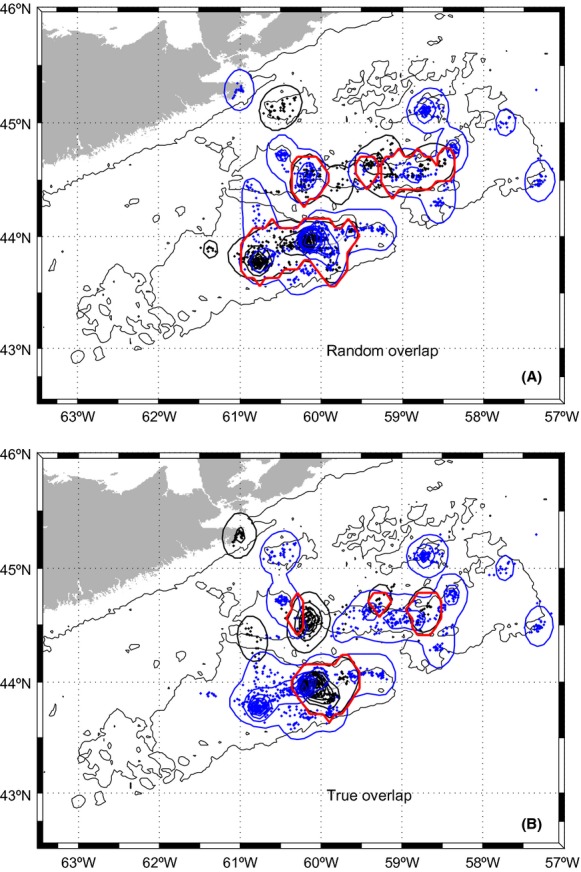
Example of one permutation of random overlap versus true overlap of kernel densities, in this case comparing adult females (black) to YOY (blue) in July. Red contour indicates the overlap of the 98.5% density area between the two kernels. Panel (A) shows the true overlap of the two groups, while (B) shows the overlap when tracks are assigned to each group randomly. Overlap of 1000 permutations of group assignment was compared to true overlap to assess if true overlap was smaller than would be produced by chance given the sample available.

A randomization analysis was used to test the null hypothesis that kernel overlap in a given monthly pairwise comparison was no larger than expected by chance given the sample available. To create a null test statistic, tracks were randomly assigned to two groups after the real overlap had been calculated for that month. Tracks were randomly shuffled by individual seal (as opposed to individual point) so that the appropriate sample sizes and individual random effect were imposed. The two groups were composed of the same number of tracks as the real observations (Pesarin 2001). Kernel densities were calculated for each of the two random groups, and the size of the overlap of their respective 98.5% density contours used as the null test statistic (Fig. 1). The random assignments were permuted 1000 times without replacement. *P*-values were determined by the proportion of random overlaps that were smaller than the observed overlap, so that if the observed real overlap was smaller than all 1000 randomly generated overlaps, then *P* ≤ 0.001.

For most months tested, 1–3 tracks terminated due to tag failure during the month and thus contributed an incomplete sample of points. Similarly, because we used only the inferred foraging locations and individual animals had differing ratios of foraging to transiting, each animal contributed a different number of points to the monthly kernels. We did not up- or down-weight these samples based on the differing contributions of each animal. The randomization analysis employed is fundamentally robust to such data imbalances (Pesarin 2001). Although short tracks may skew or bias individual kernel densities constructed with them, the variously sized tracks were randomly assigned to each of the two possible null kernels to create the 1000 null overlaps. If 95% of the null overlaps are still larger than the true overlap, any skew or bias introduced by the unevenness in track sample is unlikely to account for this difference, as the null overlaps were also created using the imbalanced data. Increasingly imbalanced data will not skew or bias results, but instead gradually lower the power of the analysis as the sample becomes more imbalanced. In the case of this analysis, when data become increasingly imbalanced, the overlap of the null kernels shrinks. When the imbalance is too high, the true overlap size ranks above the α = 0.05 level when compared to the null overlaps. So, even if there is segregation of habitat use, it is not detected because the imbalanced data do not provide sufficient statistical power.

To visualize differences in foraging areas as calculated from kernel densities, we took advantage of the normalized property of kernels. After normalizing, the volume under a 3-d kernel density surface equals one regardless of how many points were used to construct it. Given this, subtracting a kernel from itself will produce a perfectly flat surface equal to zero everywhere. Subtracting different kernels from each other will produce a surface with positive and negative regions which will vary depending upon how habitat usage differed between the groups.

In the case of kernel A minus kernel B, positive regions will indicate where habitat use was high for the population of animals used to construct kernel A and low for the population represented by kernel B; negative regions would indicate the opposite. Regions near zero indicate where habitat use was similar in both A and B. The resulting 3-d surface can then be contoured to highlight habitats that were used differentially between two groups of animals. We term this surface the “kernel density anomaly.” Because journal space is limited, we did not visualize kernel density anomalies for every month, but instead calculated and plotted anomalies by season: summer (July–September), autumn (October–December), and winter (January–April); monthly kernel density anomaly plots are, however, provided in Figs. S1–S10. The kernel density overlap analysis and the kernel density anomaly figures were produced using custom scripts in MATLAB Version 7.13 (MATLAB 2011) and visualized using the M Map mapping toolbox (Pawlowicz 2011). Working MATLAB code for the kernel overlap analysis, as well as sample data from August, is included as electronic supplement.

## Results

### Spatial distribution and habitat use

**Figure 2:**
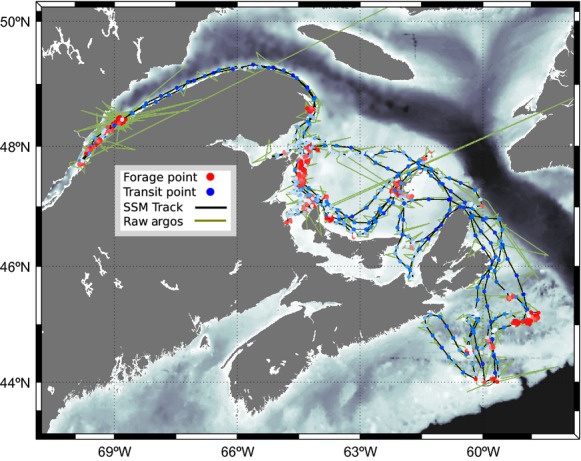
Example behavior-switching SSM model fit to a two-year-old subadult gray seal tracked by the Argos satellite network. This animal was outfitted with an Argos PTT tag manufactured by the Sea Mammal Research Unit in June 2004 and was tracked until May 2005.

SSM model diagnostics (MCMC convergence, Rhat values, etc.) suggest good model performance and reasonable behavioral inference for all tracks reported here (SSM diagnostics are reported in the appendices of Breed et al. 2009, 2011a). Older tracks with large data gaps (multiple gaps >2 days, and/or high spatial error) did not perform as well and were subsequently removed from the analysis (see Breed et al. 2011b for a careful analysis of the effect of data quality on location estimation and behavioral inference). The proportion of at-sea behaviors inferred as foraging, transiting, or uncertain varied dynamically through the year and by demographic group and was also subject to variation between individuals; these results are explored in depth in Breed et al. (2009, 2011a). An example SSM fit to an Argos track of a juvenile gray seal is shown in Fig. 2.

Results of the randomization tests revealed no significant differences between the monthly utilization distributions of male and female YOY (Table 2). Therefore, the sexes were combined into a single sample of YOY for comparison with adults.

**Table 2:** Kernel density overlap randomization results of three demographic comparisons

	YOY sexual segregation			YOY versus Adult Females			YOY versus Adult Males		
			
Month	% Overlap	Random	*P*	% Overlap	Random	*P*	% Overlap	Random	*P*
Jun	34.5	33.6 ± 0.3	0.21	33.1	34.5 ± 0.4	**0.052**	26.4	32.3 ± 0.4	**0.050**
Jul	38.4	41.5 ± 0.3	0.24	17.6	34.1 ± 0.3	**0.001**	33.3	36.5 ± 0.3	0.798
Aug	36.0	35.7 ± 0.4	0.06	16.0	35.7 ± 0.3	**0.024**	23.0	34.7 ± 0.3	0.690
Sep	24.8	30.5 ± 0.4	0.08	26.9	32.4 ± 0.3	0.124	21.6	34.8 ± 0.4	0.133
Oct	40.3	35.0 ± 0.4	0.38	32.7	44.6 ± 0.4	**0.003**	26.7	38.5 ± 0.3	0.319
Nov	31.4	31.5 ± 0.4	0.26	34.1	47.9 ± 0.4	**0.048**	15.4	37.4 ± 0.3	**0.006**
Dec	36.8	36.2 ± 0.4	0.44	32.1	33.5 ± 0.3	0.223	30.0	36.1 ± 0.4	**0.047**
Jan	11.9	17.8 ± 0.3	0.17	5.1	31.8 ± 0.4	**<0.001**	9.7	33.0 ± 0.3	**<0.001**

Overlaps were divided by the area of the larger home range (bounded by the 98.5% contour) to create a normalized percentage.

Bold values indicate statistically significant habitat segregation.

**Figure 3:**
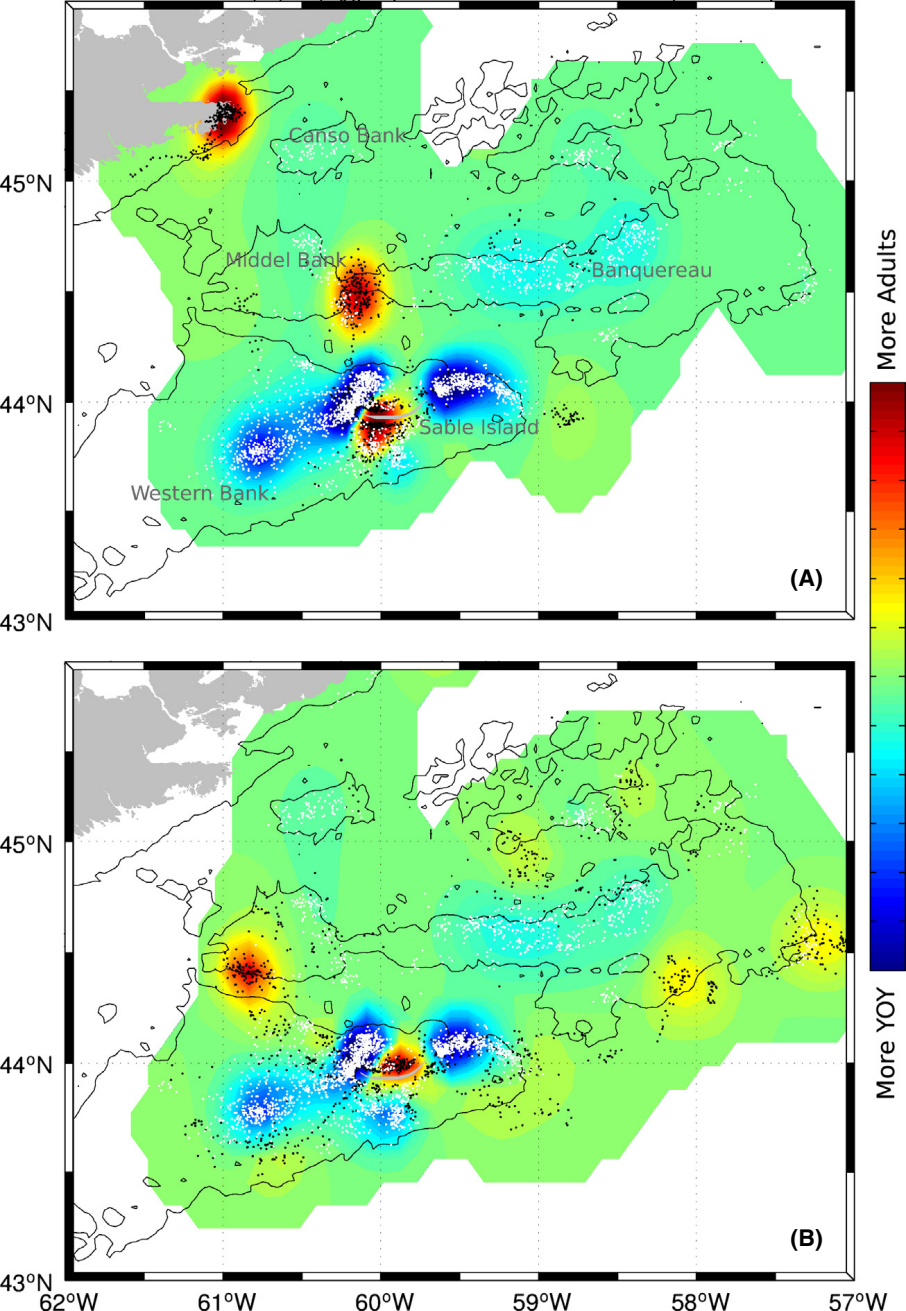
July to September kernel density anomaly for (A) adult females versus YOY and (B) adult males versus YOY. White points are SSM foraging locations of YOY, while black points are foraging locations from adults. Blue points indicate areas used more heavily by YOY, while yellow and red points indicate regions used more heavily by adults. Green areas were used equally, while white were used by neither group. Kernel density anomaly plots for each month (as opposed to season) are available in Figs. S1–S10.

**Figure 4:**
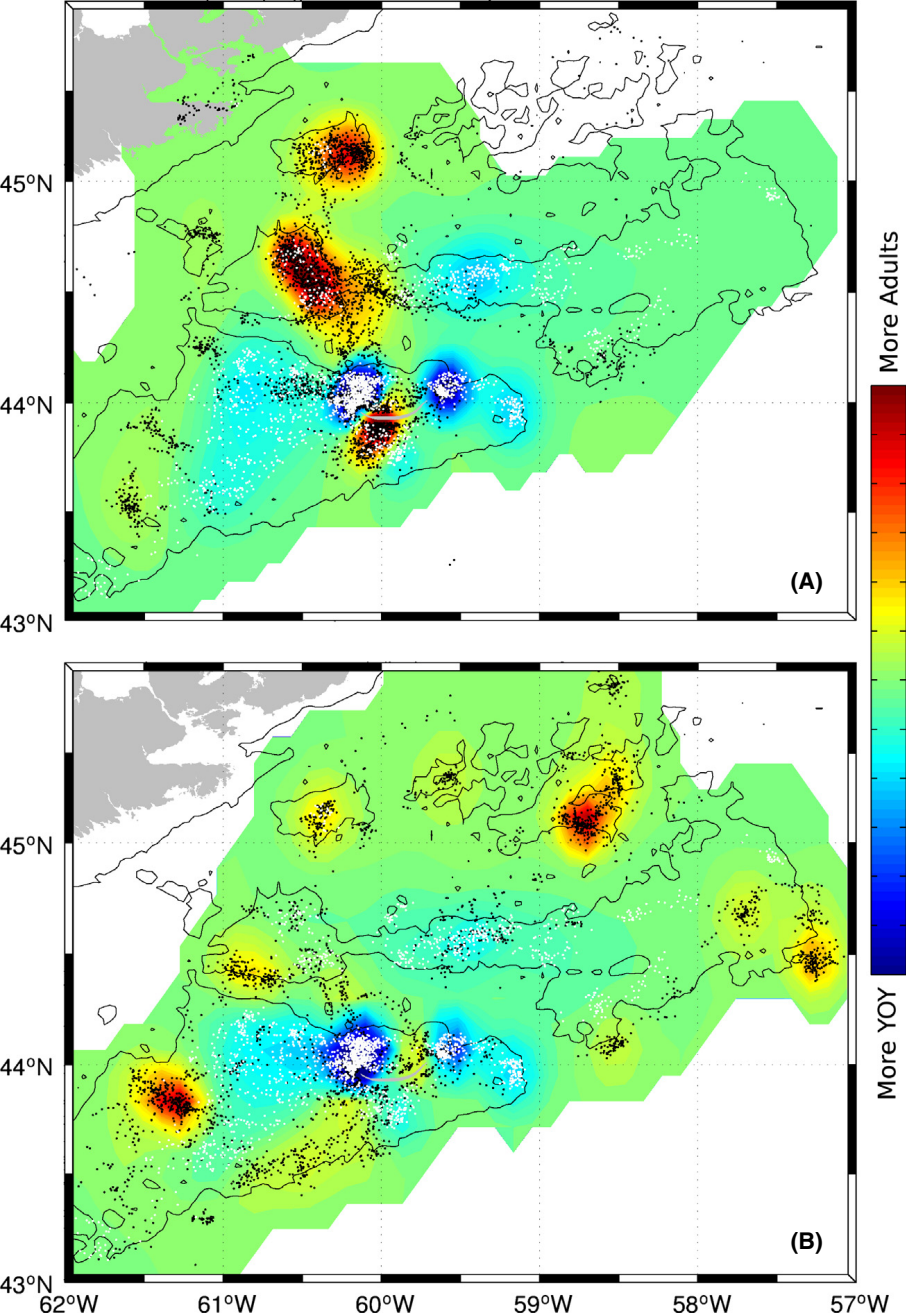
October to December kernel density anomaly for (A) adult females versus YOY (B) and adult males versus YOY. See Fig. 3legend for explanation of color.

**Figure 5:**
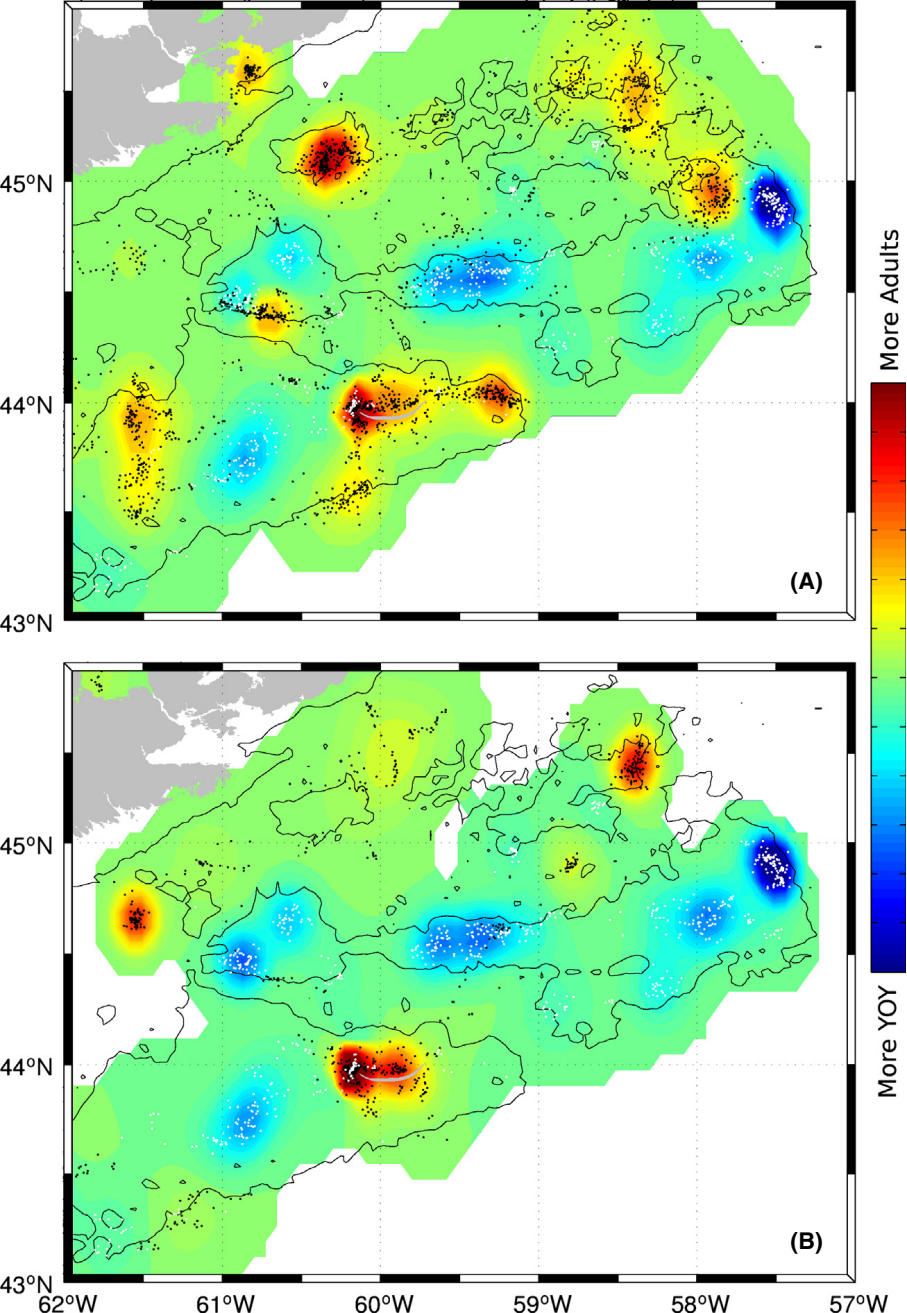
January to April kernel density anomaly for (A) adult females versus YOY and (B) adult males versus YOY. See Fig. 3legend for explanation of color.

Comparisons of YOY with adults indicate significant segregation of space use (Table 2). Although their distributions differed only marginally in June, areas used by YOY and adult females overlapped significantly less than expected during July, August, October, November, and January. Segregation was most pronounced in the summer and fall, when YOY used areas off the tips of Sable Island, Banqreau, and Western Bank, while adult females occupied areas immediately adjacent to Sable Island and the tops of the nearby Middle and Canso Banks (Figs. 3A, 4A, 5A). Spatial overlap between YOY and adult males was less than expected during June and also from November to January (Table 2). Adult males also occupied areas adjacent to Sable in summer, but not as much as adult females, and used areas near the continental shelf break more than YOY (Figs. 3B, 4B, 5B).

The winter pattern of habitat use differed markedly from that during summer and fall. YOY did not use regions near Sable Island and instead foraged over offshore banks scattered across the northern half of the Scotian Shelf (Fig. 5A,B). For all groups, winter foraging locations tended to be farther from Sable Island and became more diffuse—animals both spread out as they move away from Sable and also forage in less discrete patches than other seasons.

### Dive depths and time budgets

**Figure 6:**
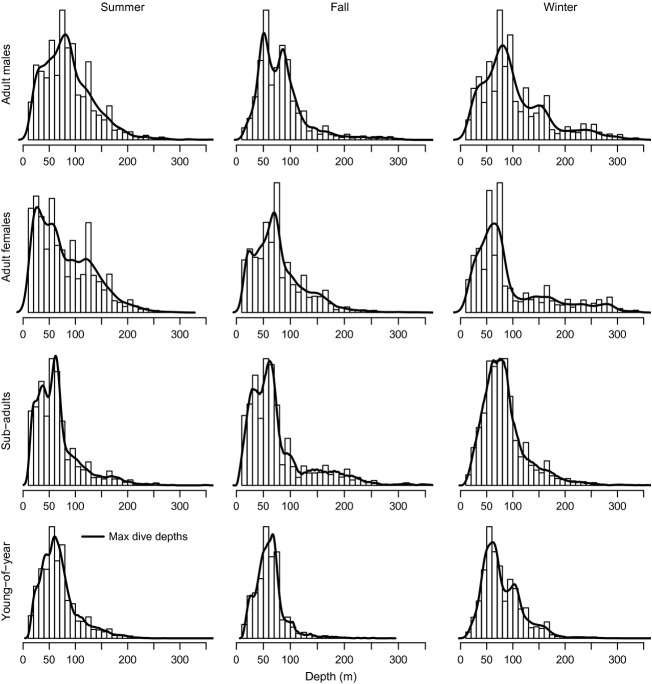
Probability density histograms and kernel densities (black line) of maximum depths reached during each 3-h bin from the onboard TDR of tags deployed in 2004 (8 adult females, 7 adult males, 24 YOY, and 6 subadults).

On average, adults made deeper dives than YOY, but owing to a large number of shallow dives made by all demographic classes, the means of the entire dive-depth distribution were not significantly different (Fig. 6, Table 3). However, when examining only the tails of the dive-depth distributions (dives >150 m), adults clearly make many more deep dives than YOY. When mixed-effects models for dive bouts are adjusted to only examine bins with dives deeper than 50 m, these differences become statistically detectable and are strongly significant in summer and fall (Table 3). The differences disappear in winter either because YOY dive performance has improved with age (Noren et al. 2005; Bennett et al. 2010), a strong ecological driver intercedes, or both. Additionally, YOY average dive durations were half that of adults year-round, a strongly significant difference (Table 3). Overall, YOY made significantly shorter and shallower dives than adults. Subadult dive-depth profiles and mean dive durations were intermediate between YOY and adults (Table 3, Table S1).

**Table 3:** Mixed-effects analysis of three key dive parameters by demographic groups

	Females	Males	Subadults	YOY
**Average Duration (min)**				
Summer	5.3 ± 0.4	4.9 ± 0.6	3.2*** ± 0.4	2.0*** ± 0.3
Fall	5.1 ± 0.3	4.7 ± 0.4	3.4*** ± 0.3	2.1*** ± 0.2
Winter	4.9 ± 0.4	3.6* ± 0.4	4.2 ± 0.3	2.5*** ± 0.2
**Max Depth (m)**				
Summer	49.4 ± 5	69.4 ± 12	53.5 ± 8	43.8 ± 7
Fall	60.1 ± 7	71.5 ± 12	54.6 ± 8	47.5 ± 6
Winter	68.0 ± 7	73.7 ± 15	77.5 ± 15	71.5 ± 9
**Max Depth bins >50 m**				
Summer	91.8 ± 6	92.0 ± 11	80.6 ± 9	70.8** ± 6
Fall	83.9 ± 5	85.2 ± 8	85.5 ± 9	69.4* ± 5
Winter	90.9 ± 7	109.9 ± 15	89.2 ± 12	83.1 ± 8

Differences between groups within each season were statically compared. *, **, and *** denote significant difference from adult females (the reference group) at the *P* < 0.05, *P* < 0.01, *P* < 0.001 levels, respectively. Models were fit with a log link, and the estimated parameters shown here have been back-transformed by exponentiation. A version of these results where parameters are estimated by month instead of season is available in Table S1.

**Figure 7:**
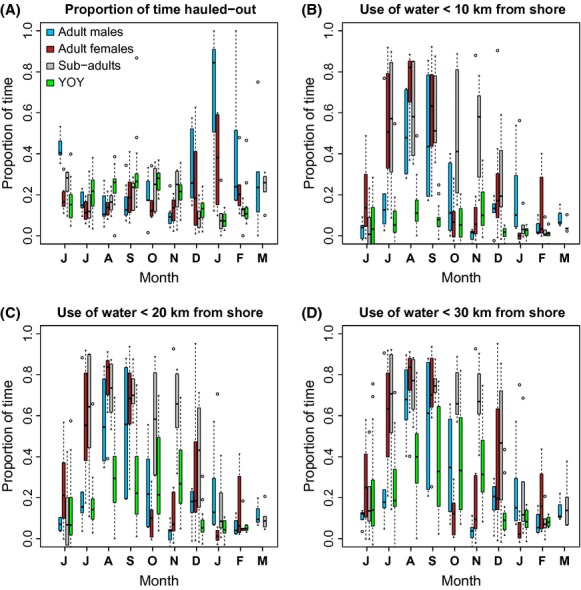
Box and whisker plots of monthly time budgets for haulout (A) and use of water within 10 km (B), 20 km (C), and 30 km (D) of shore. Negative values are possible in (B–D) because error in location observation introduced noise. This occasionally caused onshore, hauled out (as observed by wet/dry switches) to be observed as offshore.

Haulout and nearshore time budgets also differed among adults, subadults, and YOY. Through the summer and early fall, adults consistently spent about 20% of their time hauled out (Fig. 7A). Haulout increased in December at the start of the breeding season, peaked in January, then decreased but remained elevated over the winter as compared to summer. YOY and subadults displayed less dramatic seasonal fluctuations than adults, spending 20–30% of their time hauled out in summer and early fall, 10–15% in late fall, and 5–10% in January and February (Fig. 7A).

Comparison of at-sea time budgets provided an estimate of time animals spent in shallow, coastal habitat versus offshore habitat. Adults, especially adult females, spent 50–80% of their time within 10 km of shore during summer (Tab. 4, Fig. 7B–D). Adults abruptly ended coastal habitat use in October, and from October to April approached shore only to haulout. Subadults also heavily used inshore habitats and spent more than 50% of their time at sea within 10 km of shore from July to December, moving away from shorelines in January. Unlike adults or subadults, YOY spent little or no time foraging near shore at any time of year (Table 4, Fig. 7B–D), a strongly significant difference compared with adult time budgets. Finally, during the summer, YOY spent significantly more time in the band between 10 and 30 km of shore than adults (Table 4). This corroborates the kernel overlap analysis and suggests YOY either do not prefer or are displaced from waters within 10 km of shore to habitats between 10 and 30 km from shore.

**Table 4:** Time budgets for occupancy of nearshore waters (haulout excluded) by month and demographic group

	% of total time budget within 10 km of shore				% of total time budget between 10 and 30 km of shore			
		
Month	Adult Females	Adult Males	Subadults	YOY	Adult Females	Adult Males	Subadults	YOY
June	18	3	3	4	8	7	16	17
July	54	21*	55	8***	6	6	10	16†
August	74	50	60	14***	4	18	14	26††
September	60	48	58	8***	10	9	17	30††
October	9	17	48††	6‡‡	3	19	22†	32††
November	16	1	51††	13‡‡	6	3	16	24††
December	25	12	26	1**	11	7	18	11
January	7	19	5	2	5	4	19	8

Adult females, which used inshore waters to a greater degree than any other group, are the default reference group for significance comparisons. *, **, and *** denote significantly less time spent in a region than adult females at the *P* < 0.05, *P* < 0.01, *P* < 0.001 levels, respectively. Similarly, †, ††, and ††† denote significantly more time spent in a region than adult females at the *P* < 0.05, *P* < 0.01, *P* < 0.001 levels, respectively. In months when subadults used inshore regions most heavily, ‡ and ‡‡ denote significantly less time in a region than subadults at the *P* < 0.05 and *P* < 0.01 levels, respectively. Variances in these average time budgets are visualized in Fig. 7.

## Discussion

Our data clearly demonstrate a number of key behavioral differences between YOY and adult gray seals in diving behavior and habitat use. We show that YOY make shallower, shorter dives compared with adults. The shorter dives limit the benthic habitats available to YOY. We further show that foraging habitats are spatially segregated, with YOY either preferring benthic habitats farther from haulout sites or that YOY are excluded from the most accessible foraging areas near haulout sites by older animals, particularly in the summer.

The spatial segregation of foraging areas could arise from both competitive and noncompetitive ecological mechanisms. However, the perceived potential for the Sable Island gray seal population to negatively impact commercial fish stocks (Benoît et al. 2011) has led to the funding of ecological and demographic investigations of unusual intensity and duration resulting in excellent data on age-specific survival, diet, reproductive investment, and lifetime reproductive success to complement our findings on space use and movement (e.g., Austin et al. 2004; Iverson et al. 2004; Bowen et al. 2007; Lang et al. 2009; den Heyer et al. In press). We synthesize our new results within this larger body of data. Using these data, we discuss a number of potential segregating mechanisms. Although our data cannot conclusively demonstrate intraspecific competition as the segregating mechanism, we argue this hypothesis is best supported given the data available. Finally, we speculate that this competition is resulting in, or at least consistent with, compensatory density-dependent population regulation.

### Case for intraspecific competition

#### Exclusion of YOY from areas near Sable Island

Kernel anomalies, time budgets, and the randomization analysis indicate YOY avoid nearshore habitats to forage at more distant foraging patches from foraging areas near Sable Island. There are three likely reasons for this.

First, adults and YOY might feed on different prey and these prey are located in different areas around Sable Island. Other observations suggest that this is unlikely. Although YOY foraged farther from Sable, they used shallow banks to the east, west, and north that are similar to the sandy areas around the island. All age groups foraged in shallow areas and made shallow dives in the summer and early fall, suggesting a large degree of overlap in the use of shallow sandy areas and therefore prey taken. Although we have no summer diet data for subadults or YOY, during spring, individual YOY consume a wide range of prey species, suggesting a generalist foraging strategy of taking any prey item they encounter and are able to catch (Beck et al. 2007). Beck et al. (2007) also showed that adults are usually highly specialized on one or a small number of prey types. As a group, adult females consume the same broad spectrum of prey items as YOY, but they are individually specialized, so the variation is between rather than within individuals. Such specializations have been shown to lead to more efficient foraging and arise when intraspecific competition limits resources (Holbrook and Schmitt 1992; Tinker et al. 2008b).

The second potential cause of spatial segregation is differential response to predation. The large number of seals using Sable Island attract sharks, which prey on both gray and harbor seals (*Phoca vitulina*) (Brodie and Beck 1983). In winter, seals are thought to be incidentally killed by Greenland sharks (*Somniosus microcephalus*), while migratory sharks such as white sharks (*Carcharodon carcharias*) target seals as prey in the summer (Lucas and Stobo 2000). The small size of juveniles makes them more vulnerable to shark predation, and they suffer greater shark-induced mortality than adults. Vulnerable gray seal YOY may forage farther from the colony to lessen predation risk, and travel through near-shore waters around the colony quickly when moving to and from haulout. Many species balance predation risk against potential foraging success or efficiency (e.g., Mittelbach 1981; Lima and Valone 1986; Sih et al. 1998), and YOY may undertake longer trips with more transit time to avoid sharks around the colony. Thus, differential response to predation risk by YOY is another possible explanation of the observed patterns given available observations.

**Figure 8:**
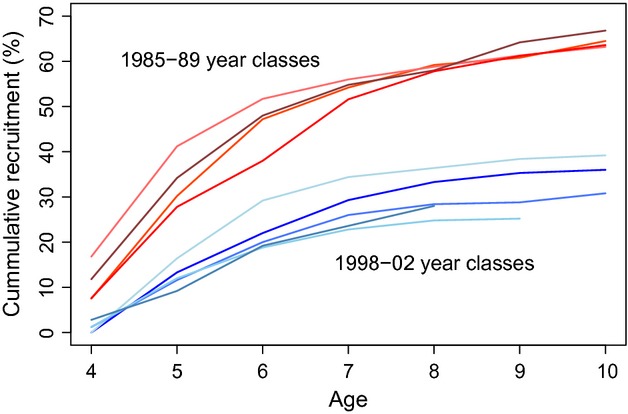
Recruitment to the breeding population by females branded from 1985 to 1989 (red and orange shades) and 1998 to 2002 (blue shades). Data from den Heyer et al. (In press).

Finally, and we believe most likely, intraspecific competition with adults, females in particular, may exclude YOY from foraging near Sable Island as well as other key foraging areas during summer and early fall. The population of gray seals breeding on Sable Island grew exponentially from 1962 into the early 2000s, with an estimated 2004 population of 159,000 animals (Trzcinski et al. 2006). The rate of increase in pup production has recently declined and is now following a logistic growth curve (Bowen et al. 2007, 2011). Most of this population feeds on the Scotian Shelf, with considerable foraging effort focused near Sable Island (Trzcinski et al. 2006; Breed et al. 2011a; this study). This large increase in population size has likely increased competition for prey. Mark–resight data from uniquely marked females indicate that animals born between 1998 and 2002 recruited into the reproductive population at half the rate of animals born in the late 1980s (Fig. 8). Over the same period, adult survival has remained high (den Heyer et al In press). These observations, combined with at-sea distribution of YOY compared with adults, suggest that YOY are being outcompeted by adults and particularly by adult females. Spatial segregation of foraging areas has been implicated as evidence of intraspecific competitions around marine breeding colonies in other species (e.g., Grémillet et al. 2004; Field et al. 2005), until this study, however, survival data have never been available to confirm if or how the apparent competition affects the population.

To understand how intraspecific competition may be responsible for the observed segregation patterns, we first need to understand seasonal fish migration patterns on the Scotian Shelf and around Sable Island. Fish migrations are caused by seasonal changes in water temperature, oceanography, and primary productivity (Perry and Smith 1994; Swain et al. 1998; Comeau et al. 2002). These migrations have been best documented in ground fish species, including Atlantic cod (*Gadus morhua*), haddock (*Melanogrammus aeglefinus*), silver hake (*Merluccius bilinearis*), and American plaice (*Hippoglossoides platessoides*) (Perry and Smith 1994; Swain et al. 1998). None of these species are especially prominent in gray seal diets (Beck et al. 2007), but the seasonal pattern of fish migration is pervasive and present in a large fraction of fish species on the Scotian Shelf (Horsman and Shackell 2009).

The general migratory pattern is for fish to move into warm, shallow water in the summer to forage, while wintering in deep basins where waters remain above zero (Perry and Smith 1994) to avoid freezing.

In summer, waters become warm and productive, which cause fish to move out of wintering areas and into shallow areas such as the broad, shallow, sandy platform surrounding Sable Island to forage. This migration brings prey within close proximity of the main seal colony. As they forage through the summer, fishes develop increased lipid reserves, which peak in August or September (Comeau et al. 2002). The net result of warm productive summer months is larger numbers of energy rich prey close to the population center of gray seals. The accessible prey allow animals to accumulate blubber with a relatively modest foraging effort over the summer. Thus, the summer aggregation of prey in shallow, often nearshore, water sets up an intraspecific competition for these easily accessible resources.

In late fall and early winter, all ages of gray seals move foraging effort far offshore, likely following the return migrations of their prey into deeper waters along shelf edges and into shelf basins where water remains warm. Lipid reserves of prey also gradually become depleted through the winter, and individual prey captures are thus less energetically rewarding (Smigielski et al. 1984; Robards et al. 1999; Comeau et al. 2002; Kitts et al. 2004; Rosen and Trites 2004). So although the winter prey distribution relaxes intraspecific competition, prey are less accessible and of lower quality so that foraging gray seals of all ages must rely on blubber reserves accumulated during the summer and fall to comfortably survive the winter. If YOY are accumulating less blubber during the summer because they are displaced from areas where prey are most accessible and/or of high quality, they consequently incur decreased winter survival. We believe this is the action responsible for the complex patterns of space use and survival observed in Sable Island gray seals over the past 20 years.

#### Compensatory versus overcompensatory population regulation in colonially breeding marine animals

If intraspecific competition is affecting YOY in the way we hypothesize with YOY physically displaced from key summer and fall foraging areas and the lost foraging opportunities causing increased mortality, then we may draw some further inferences about how the population dynamic is being affected by these interactions. Male and female adults, as well as subadults, are experiencing neither increased mortality nor displacement. Thus, the competition is producing a clear loser. As there are clear winners and losers, winning competitors receive an adequate ration while losing competitors do not, the competition should lead to compensatory density-dependent population regulation (Nicholson 1954; May et al. 1974; Turchin 2003). This should lead to a gradual approach to the population's carrying capacity, which is consistent with census data (Bowen et al. 2007, 2011), and also gray seal population trajectories in Scotland (SCOS 2011).

Although gray seals and some other phocid seals are capital breeders (Schulz and Bowen 2005), which provide energy for their offspring entirely based on stored reserves, most colony-breeding marine animals are income breeders (e.g., penguins, sea lions, fur seals, alcid and procellariiform sea birds). Income breeding species acquire resources to provision–dependent young by making repeated foraging trips during the provisioning period. During this period of offspring dependence, parents are central-place foragers and restricted to foraging nearby so they may regularly provision young. They must also meet their own energetic needs, which, if resource patches are distant, can dominate the resources acquired during foraging trips leaving few resources for dependent young (e.g., Arnould et al. 1996; Boyd 1999). These limitations should lead to scramble competition among adults foraging to provision young in areas around colonies. Theoretically, in a scramble competition, once a resource becomes limiting the population reaches a break point, and no individuals are able to acquire sufficient resources (Nicholson 1954; Turchin 2003). This leads to sudden, large increases in mortality. Such mass reproductive failures are often observed in income breeding otariids and seabirds (Trillmich and Limberger 1985; Trites and Donnelly 2003; Wanless et al. 2005). However, truly catastrophic overcompensatory collapses rarely occur. Females may abandon offspring when they cannot collect enough resources. At abandonment, females move away from the colonies to lessen competition. This relaxes the intraspecific competition around the colony and greatly dampens the overcompensatory effect (Satterthwaite and Mangel 2012). Still, year-to-year offspring survival at colonies of fur seals and sea lions is much more volatile than observed in Sable Island gray seals.

The functional response of density dependence in this gray seal population is compensatory due, at least in part, to a capital-breeding life history affecting the nature of intraspecific competition near K. In addition to increased mortality of juveniles as the population approaches K, we expect a gradual lowering of fecundity in adult females, gradual decrease in adult survival, and a smooth approach to carrying capacity. The colony of gray seals breeding at Sable Island is ecologically and behaviorally similar to fur seal and sea lion colonies elsewhere, but the life-history difference (capital vs. income breeding) induces a different functional response to density dependence, which in turn causes population stability and resilience to year-to-year changes in prey availability. Such a stable population of predators would be expected to induce ecosystem stability (Heithaus et al. 2008) and require a different management plan compared with other colony-breeding marine animals.

## Supporting Information

**Data S1.** Complete Matlab code, instructions, and sample data to run the kernel overlap randomization analysis.

**Figure S1.** Kernel density anomaly plot for June. Cold colors and white points represent YOY; warm colors and black points adults. Panel (A) adult females versus YOY, Panel (B) adult males versus YOY.

**Figure S2.** Kernel density anomaly plot for July. Cold colours and white points represent YOY; warm colours and black points adults. Panel (A) adult females versus YOY, Panel (B) adult males versus YOY.

**Figure S3.** Kernel density anomaly plot for August. Cold colours and white points represent YOY; warm colours and black points adults. Panel (A) adult females versus YOY, Panel (B) adult males versus YOY.

**Figure S4.** Kernel density anomaly plot for September. Cold colours and white points represent YOY; warm colours and black points adults. Panel (A) adult females versus YOY, Panel (B) adult males versus YOY.

**Figure S5.** Kernel density anomaly plot for October. Cold colours and white points represent YOY; warm colours and black points adults. Panel (A) adult females versus YOY, Panel (B) adult males versus YOY.

**Figure S6.** Kernel density anomaly plot for November. Cold colours and white points represent YOY; warm colours and black points adults. Panel (A) adult females versus YOY, Panel (B) adult males versus YOY.

**Figure S7.** Kernel density anomaly plot for December. Cold colours and white points represent YOY; warm colours and black points adults. Panel (A) adult females versus YOY, Panel (B) adult males versus YOY.

**Figure S8.** Kernel density anomaly plot for January. Cold colours and white points represent YOY; warm colours and black points adults. Panel (A) adult females versus YOY, Panel (B) adult males versus YOY.

**Figure S9.** Kernel density anomaly plot for February. Cold colours and white points represent YOY; warm colours and black points adults. Panel (A) adult females versus YOY, Panel (B) adult males versus YOY.

**Figure S10.** Kernel density anomaly plot forMarch. Cold colours and white points represent YOY; warm colours and black points adults. Panel (A) adult females versus YOY, Panel (B) adult males versus YOY.

**Table S1.** Max dive depth, Max dive depth of bins with dives 50 m, and mean dive duration, estimated for each group by month (versus. season as in the main text).
