# Supplementary material for: Behavioral signature of intraspecific competition and density dependence in colony-breeding marine predators
Source: Ecol Evol. 2013 Sep 12;3(11):3838–54. doi: 10.1002/ece3.754 (PMC3810878; doi:10.1002/ece3.754)
Supplement: Supplementary file 1 [file ece30003-3838-SD1.pdf]

Appendix 1: Supplemental Figures & Tables

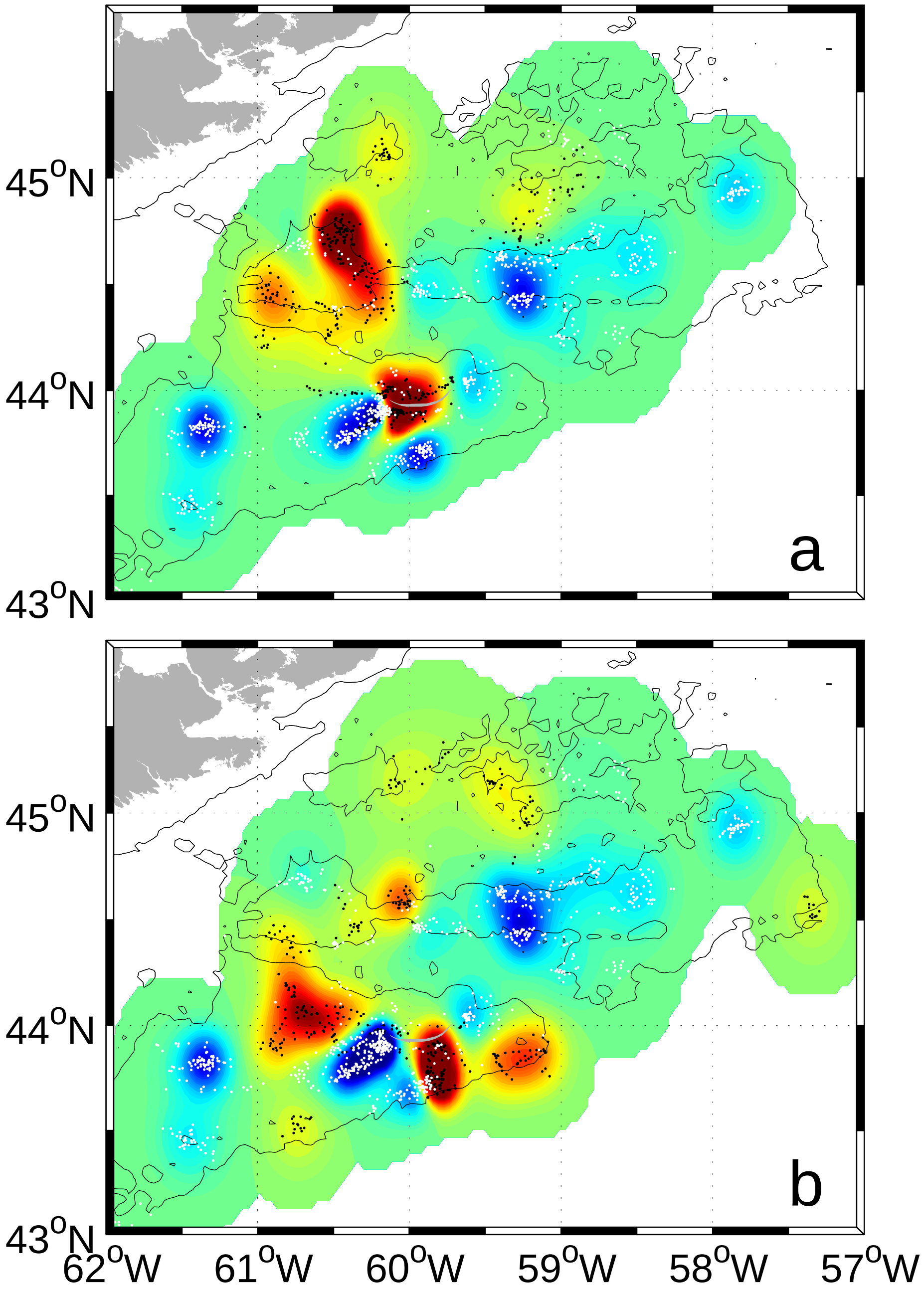

Figure A-1. Kernel density anomaly plot for June. Cold colors and white points represent YOY; warm colors and black points adults. Panel a) adult females vs YOY, Panel b) adult males vs YOY.

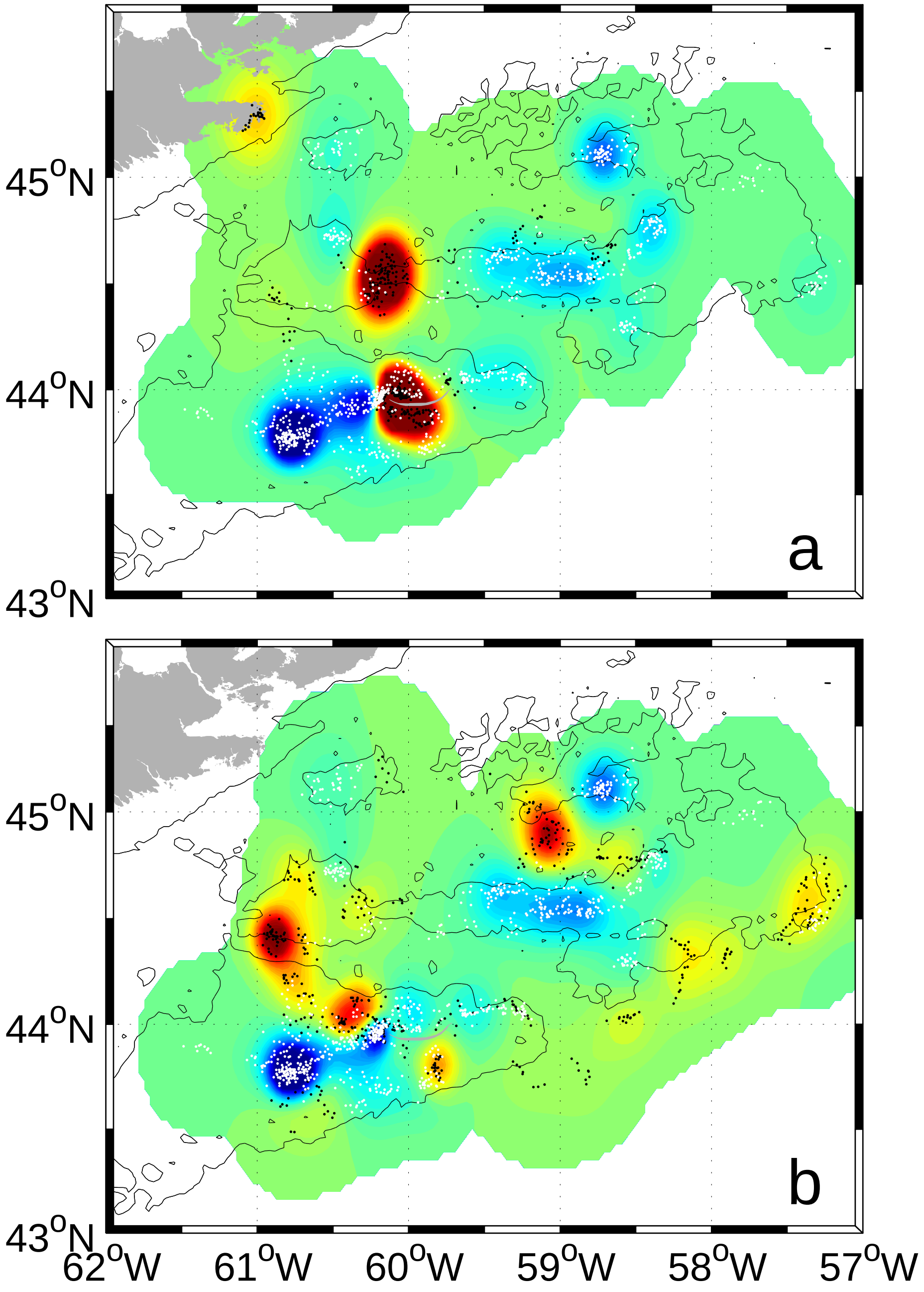

Figure A-2. Kernel density anomaly plot for July. Cold colours and white points represent YOY; warm colours and black points adults. Panel a) adult females vs YOY, Panel b) adult males vs YOY.

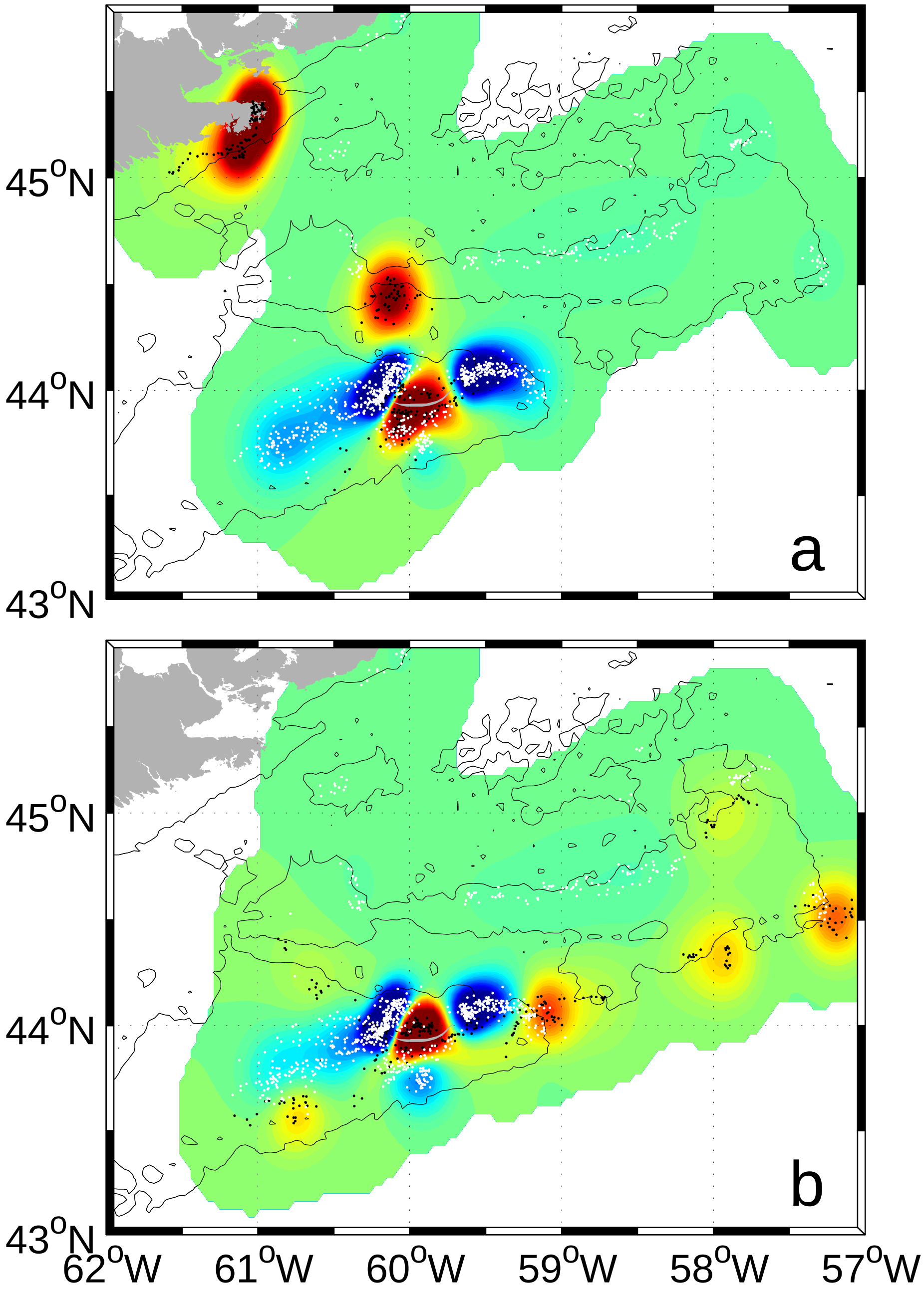

Figure A-3. Kernel density anomaly plot for August. Cold colours and white points represent YOY; warm colours and black points adults. Panel a) adult females vs YOY, Panel b) adult males vs YOY.

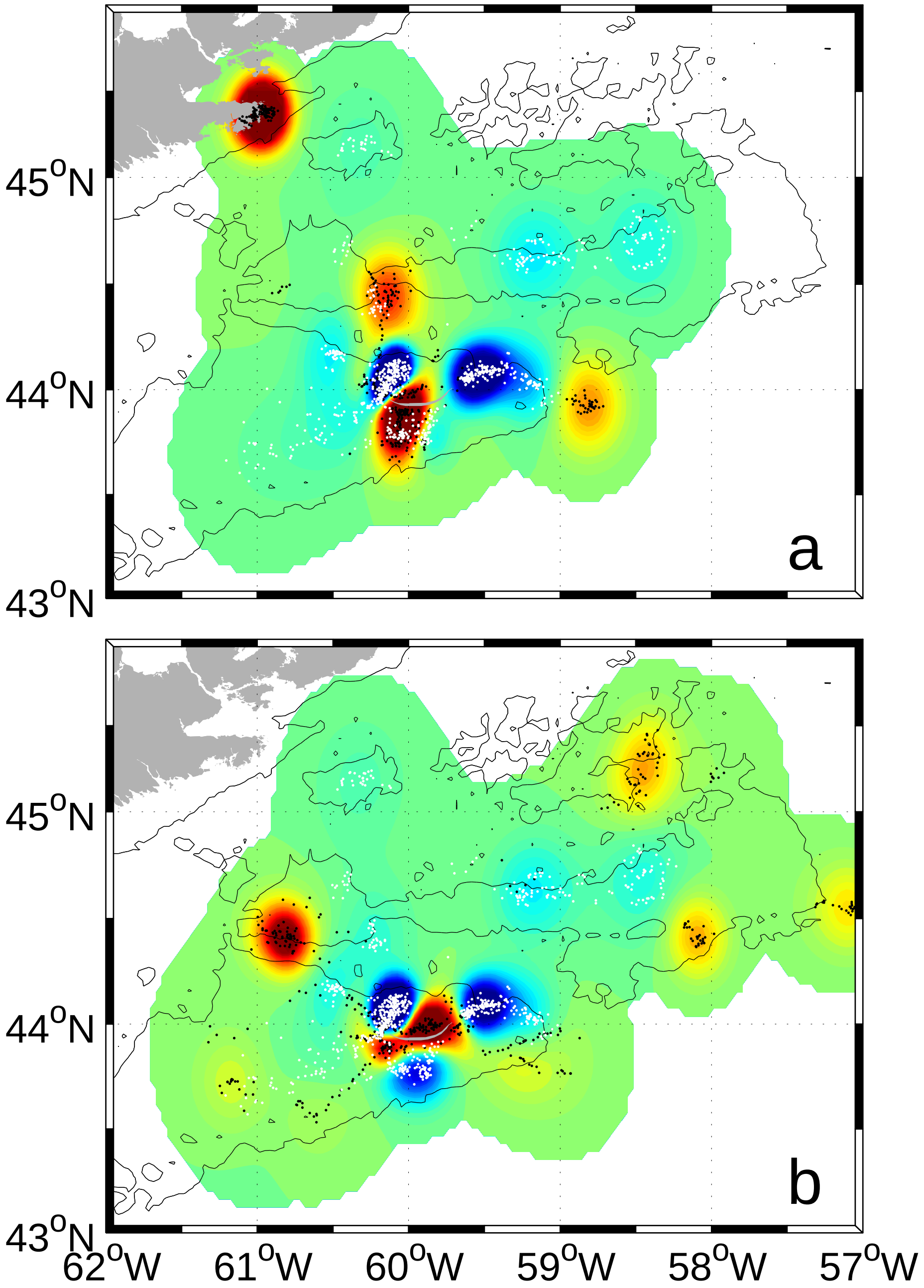

Figure A-4. Kernel density anomaly plot for September. Cold colours and white points represent YOY; warm colours and black points adults. Panel a) adult females vs YOY, Panel b) adult males vs YOY.

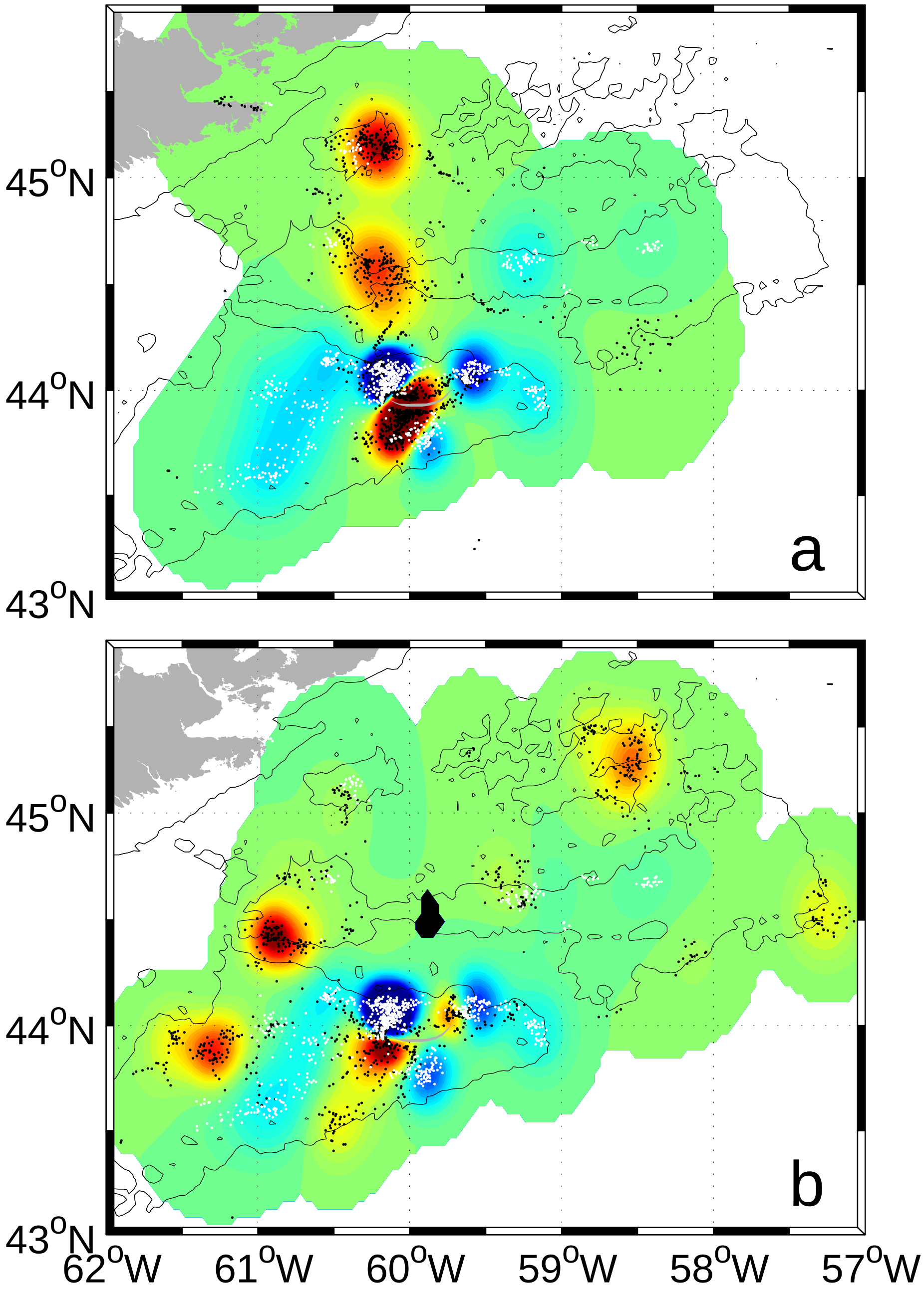

Figure A-5. Kernel density anomaly plot for October. Cold colours and white points represent YOY; warm colours and black points adults. Panel a) adult females vs YOY, Panel b) adult males vs YOY.

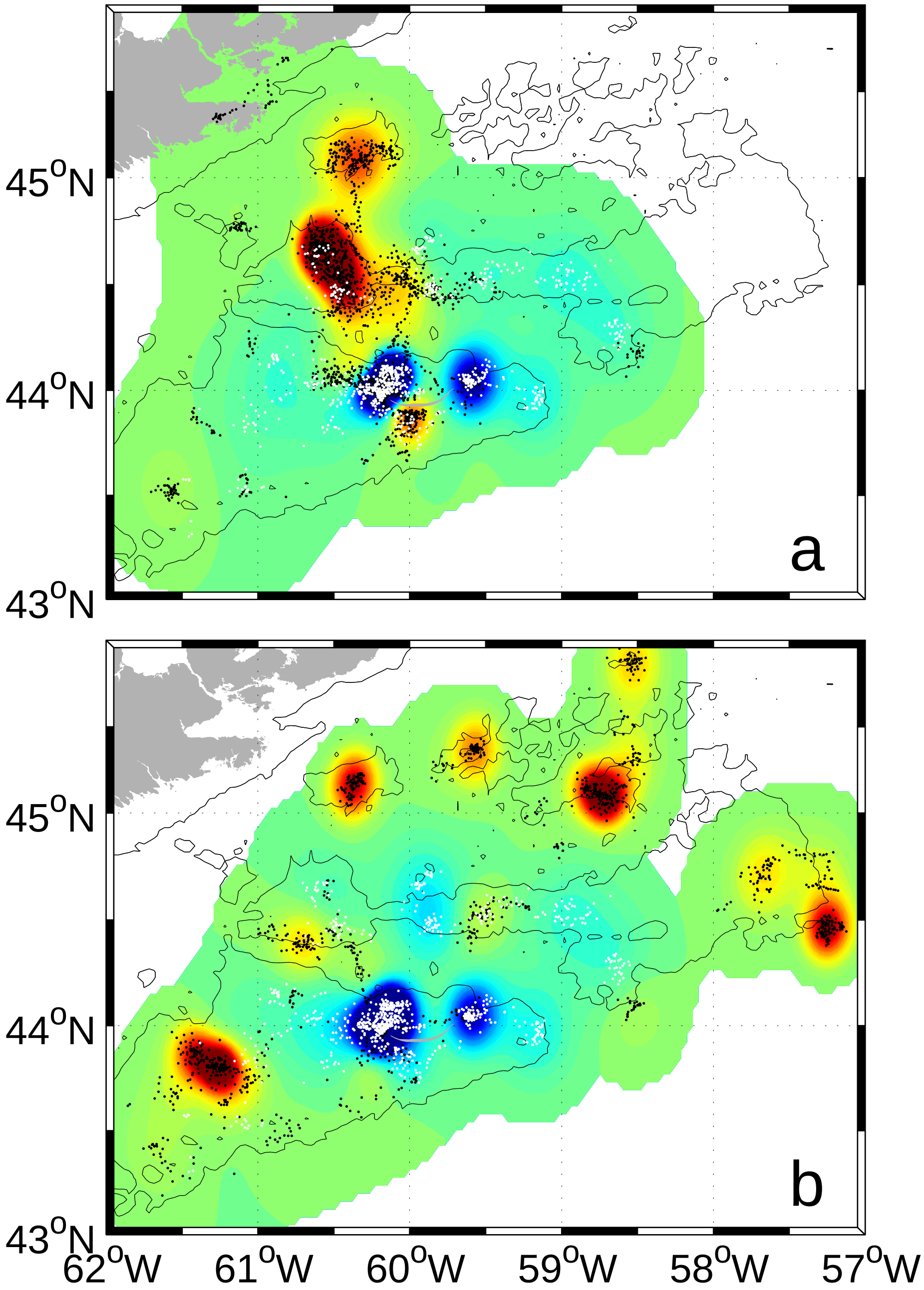

Figure A-6. Kernel density anomaly plot for November. Cold colours and white points represent YOY; warm colours and black points adults. Panel a) adult females vs YOY, Panel b) adult males vs YOY.

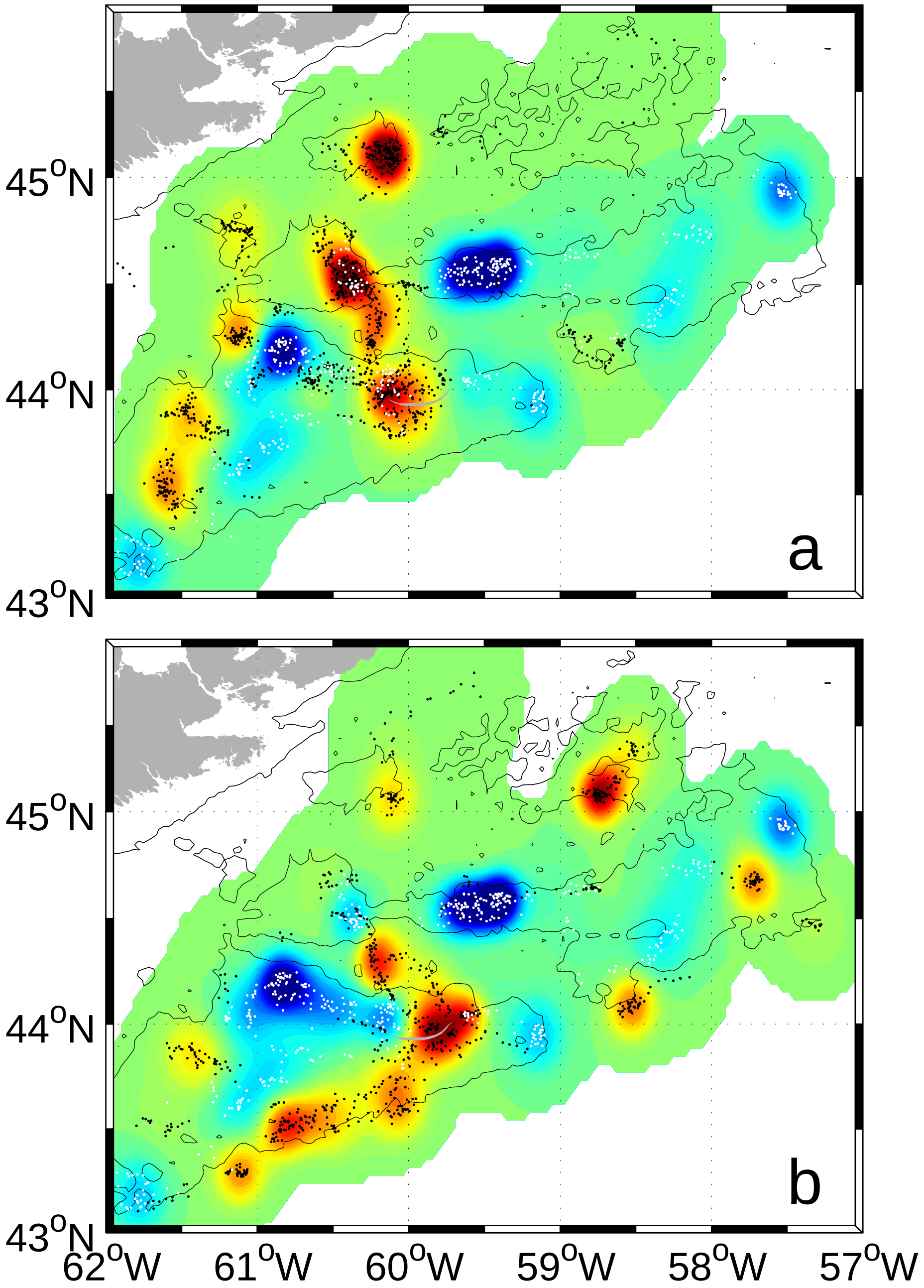

Figure A-7. Kernel density anomaly plot for December. Cold colours and white points represent YOY; warm colours and black points adults. Panel a) adult females vs YOY, Panel b) adult males vs YOY.

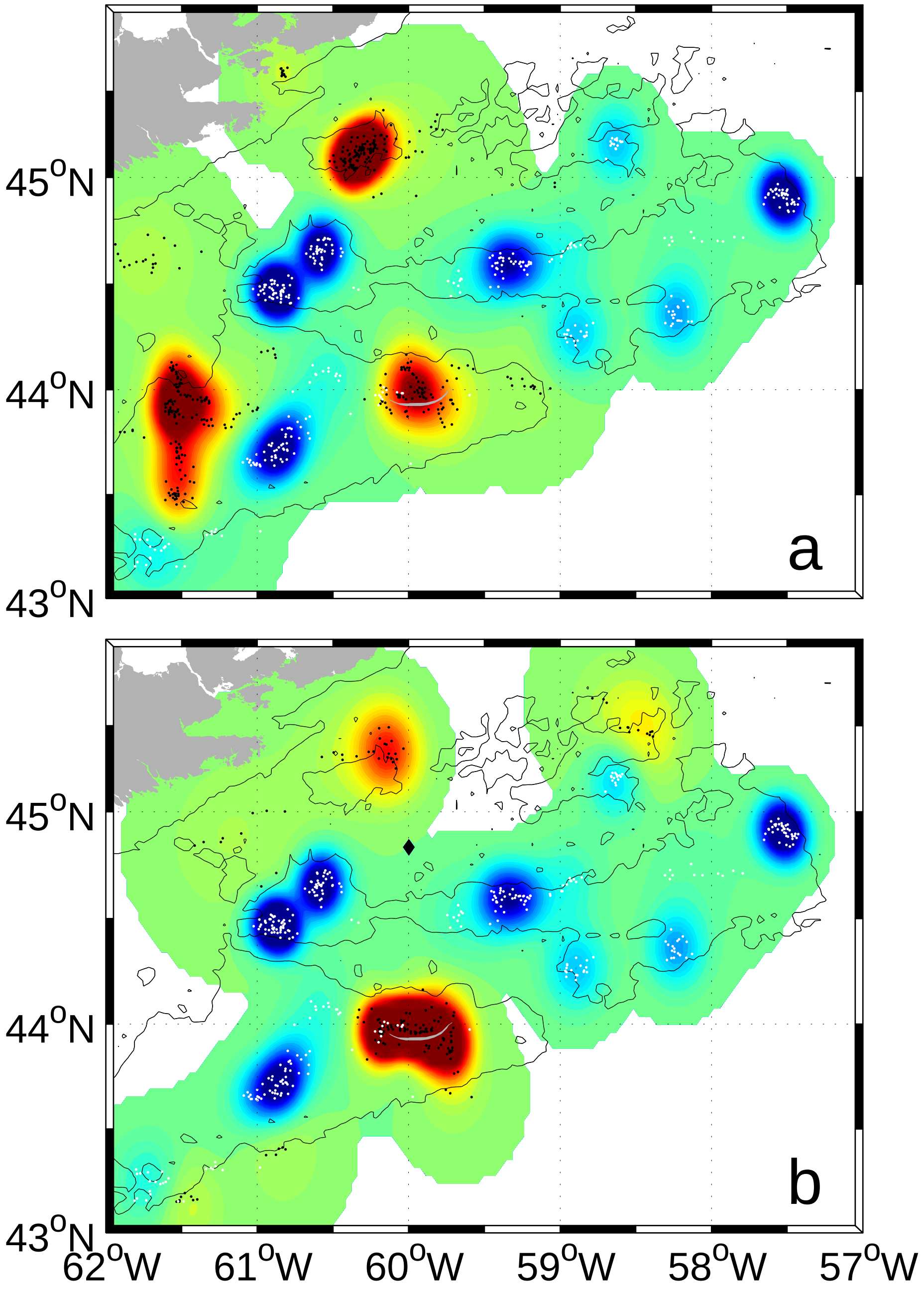

Figure A-8. Kernel density anomaly plot for January. Cold colours and white points represent YOY; warm colours and black points adults. Panel a) adult females vs YOY, Panel b) adult males vs YOY.

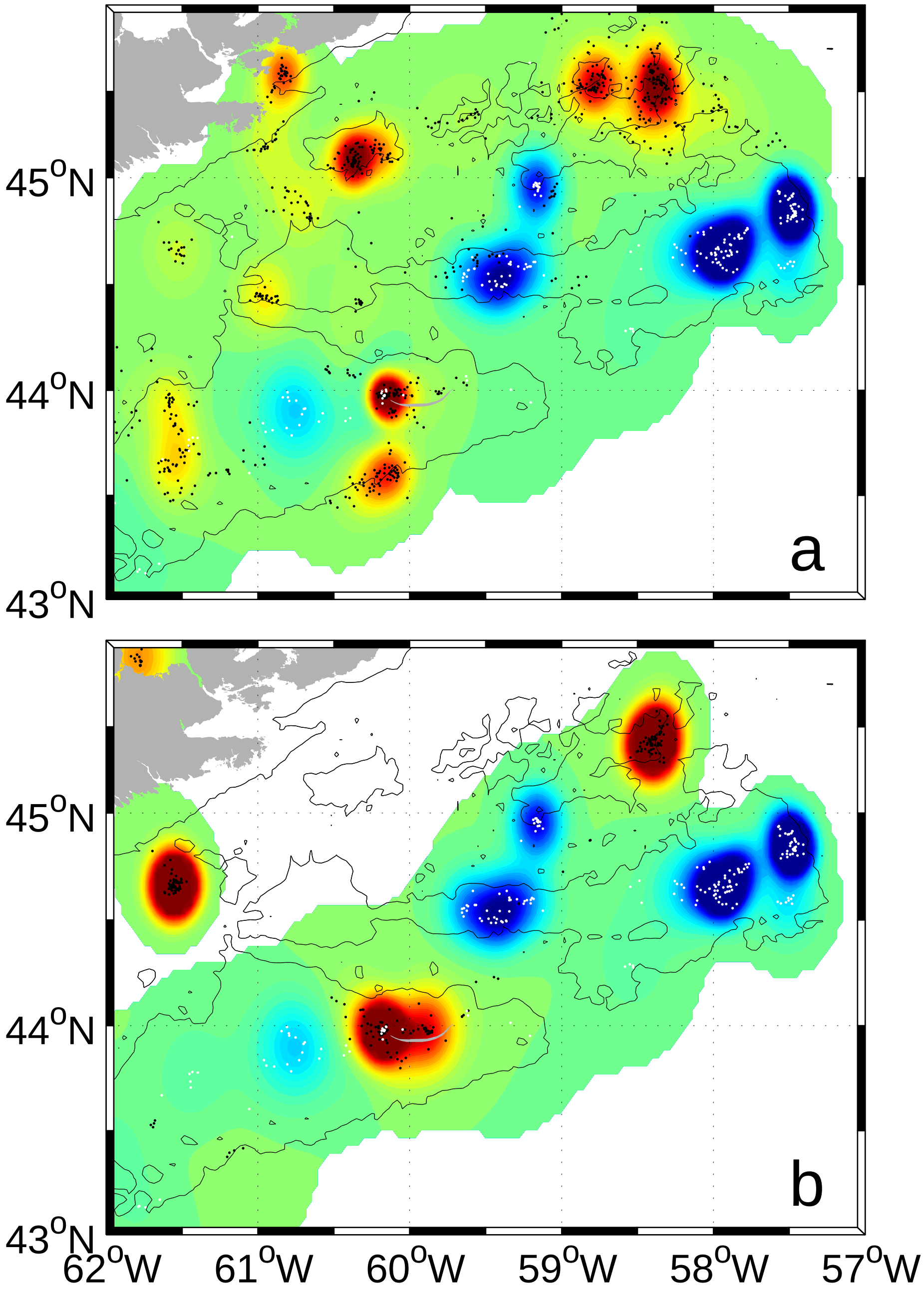

Figure A-9. Kernel density anomaly plot for February. Cold colours and white points represent YOY; warm colours and black points adults. Panel a) adult females vs YOY, Panel b) adult males vs YOY.

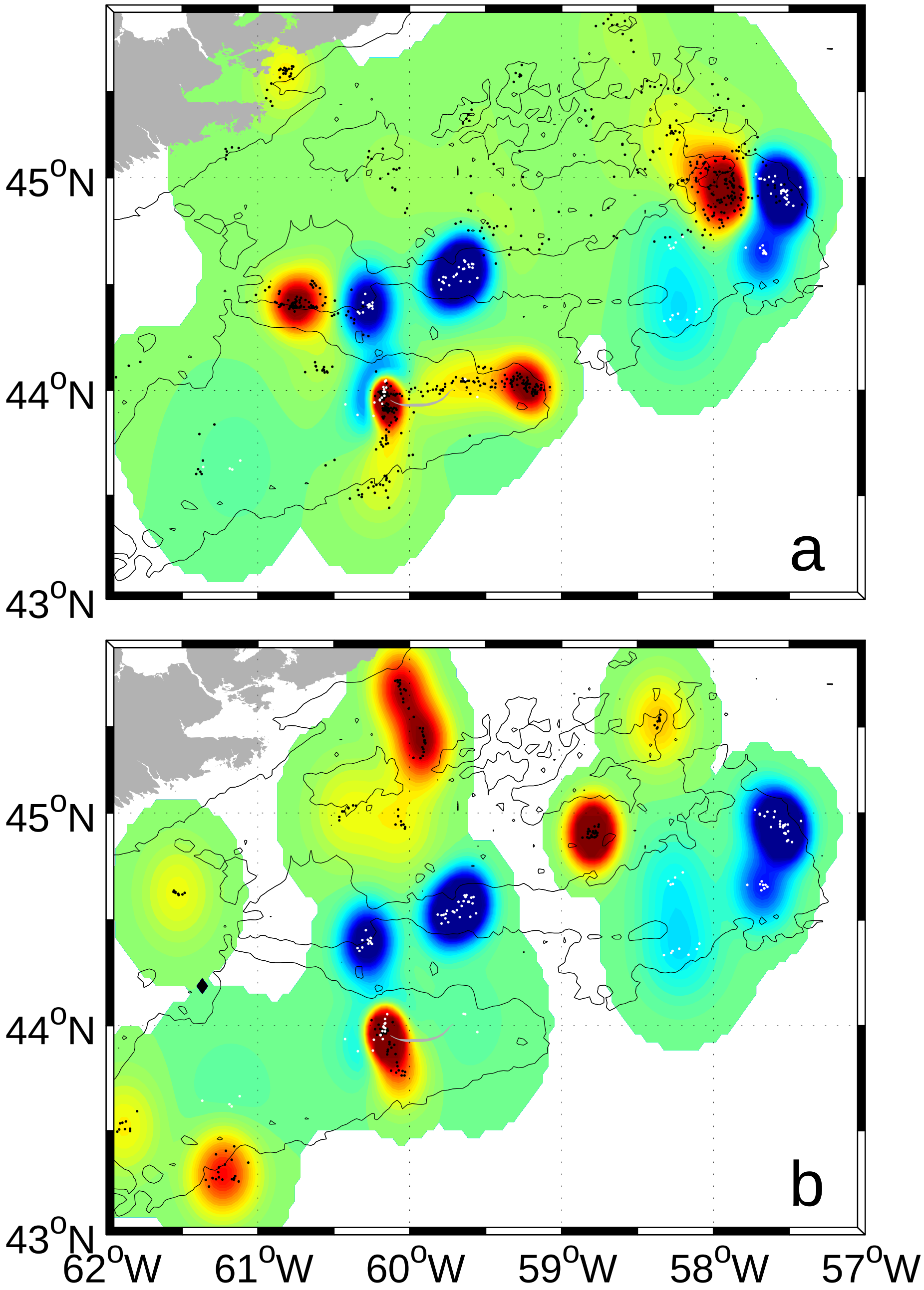

Figure A-10. Kernel density anomaly plot for March. Cold colours and white points represent YOY; warm colours and black points adults. Panel a) adult females vs YOY, Panel b) adult males vs YOY.

Table A-1. Max dive depth, Max dive depth of bins with dives > 50 m, and mean dive duration, estimated for each group by month (vs. season as in the main text).

**Average Duration (min)**	Females	Males	Sub-Adults	YOY
June	4.2 ± 0.3	3.9 ± 0.4	2.9 ± 0.3	1.9 ± 0.2
July	5.6 ± 0.4	4.6 ± 0.6	2.9 ± 0.4	2.0 ± 0.2
August	5.8 ± 0.5	5.7 ± 0.7	3.5 ± 0.4	2.2 ± 0.2
September	5.4 ± 0.5	5.4 ± 0.7	3.8 ± 0.5	2.2 ± 0.3
October	5.6 ± 0.4	5.0 ± 0.4	3.6 ± 0.4	2.3 ± 0.2
November	5.0 ± 0.3	4.7 ± 0.4	3.3 ± 0.3	2.1 ± 0.2
December	4.9 ± 0.3	4.7 ± 0.5	3.7 ± 0.4	2.4 ± 0.2
January	5.2 ± 0.4	3.3 ± 0.4	4.0 ± 0.5	2.5 ± 0.3
February	5.3 ± 0.5	4.0 ± 0.6	4.7 ± 0.7	2.6 ± 0.3
March	5.2 ± 0.4	3.8 ± 0.4	4.7 ± 0.8	2.5 ± 0.4
**Max Depth (m)**	Females	Males	Sub-Adults	YOY
June	72.4 ± 7	79.7 ± 12	61.5 ± 10	66.4 ± 8
July	54.7 ± 9	72.3 ± 17	47.8 ± 12	51.9 ± 10
August	37.3 ± 6	63.9 ± 16	52.8 ± 13	51.7 ± 10
September	38.8 ± 7	69.6 ± 18	44.3 ± 12	60.7 ± 12
October	62.5 ± 10	69.6 ± 16	48.3 ± 12	55.9 ± 10
November	61.7 ± 6	73.2 ± 26	50.4 ± 16	47.8 ± 6
December	55.1 ± 7	71.1 ± 13	64.5 ± 13	64.1 ± 10
January	78.8 ± 9	52.3 ± 10	80.0 ± 10	70.1 ± 11
February	74.7 ± 10	97.1 ± 21	83.4 ± 17	75.3 ± 13
March	46.1 ± 8	96.4 ± 29	70.0 ± 18	72.5 ± 18
**Max Depth (bins $<$ 50 m excluded)**	Females	Males	Sub-Adults	YOY
June	94.6 ± 7	93.3 ± 11	79.9 ± 10	71.1 ± 7
July	95.9 ± 8	92.0 ± 11	80.7 ± 110	70.1 ± 7
August	86.4 ± 8	93.9 ± 12	81.3 ± 10	69.2 ± 7
September	90.9 ± 9	90.3 ± 13	74.1 ± 11	73.1 ± 9
October	87.1 ± 8	86.6 ± 11	81.6 ± 12	67.4 ± 7
November	83.2 ± 7	85.6 ± 10	85.5 ± 11	67.6 ± 7
December	80.0 ± 7	82.7 ± 10	86.9 ± 12	76.3 ± 8
January	90.4 ± 8	86.8 ± 13	91.1 ± 12	82.7 ± 9
February	95.7 ± 10	117.2 ± 20	92.1 ± 15	84.6 ± 12
March	86.6 ± 11	108.3 ± 23	79.5 ± 16	83.2 ± 15
